# Supplementary figures and images for: Robust and brain-like working memory through short-term synaptic plasticity
Source: PLoS Comput Biol. 2022 Dec 27;18(12):e1010776. doi: 10.1371/journal.pcbi.1010776 (PMC9829165; doi:10.1371/journal.pcbi.1010776)

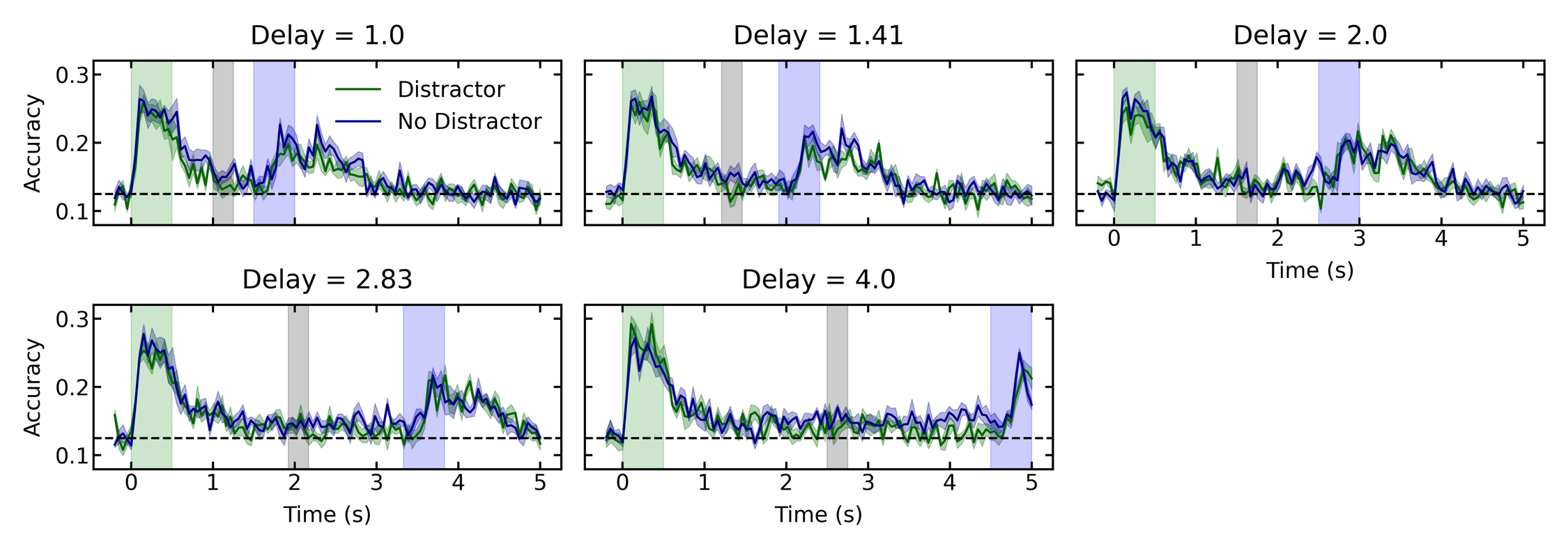

Supplement: S1 Fig — We evaluated performance on held-out data. Sample decoding accuracy drops to approximately chance during the delay. (TIF) [file pcbi.1010776.s001.tif]

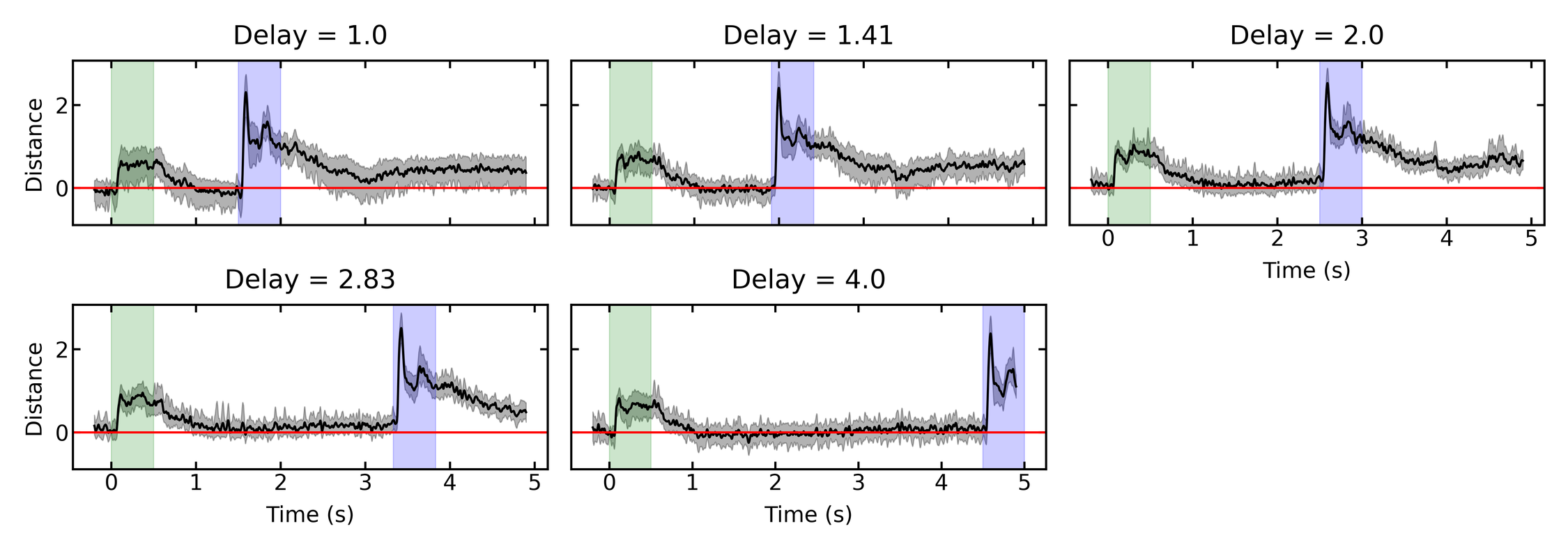

Supplement: S2 Fig — (TIF) [file pcbi.1010776.s002.tif]

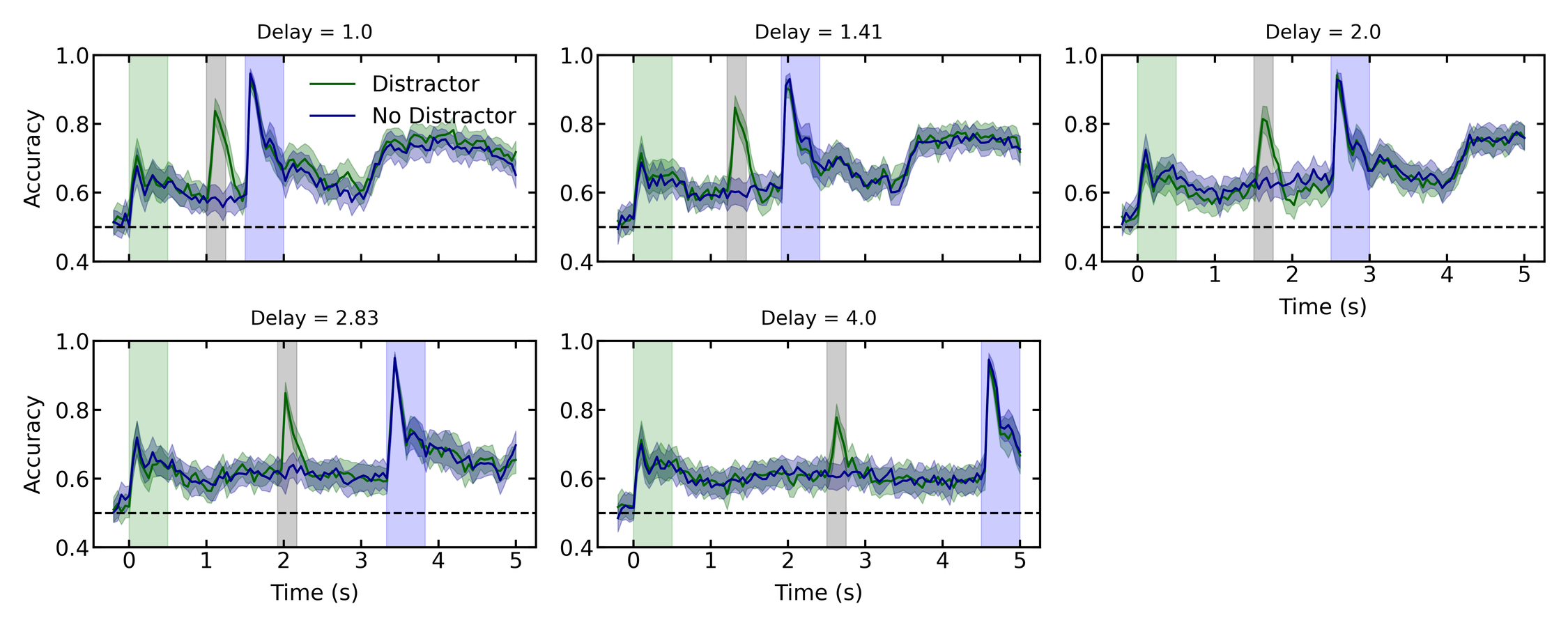

Supplement: S3 Fig — Spiking contains information about pre vs. post sample presentation through the delay. This indicates that neural spiking does not return to its pre-sample firing pattern following sample presentation. (TIF) [file pcbi.1010776.s003.tif]

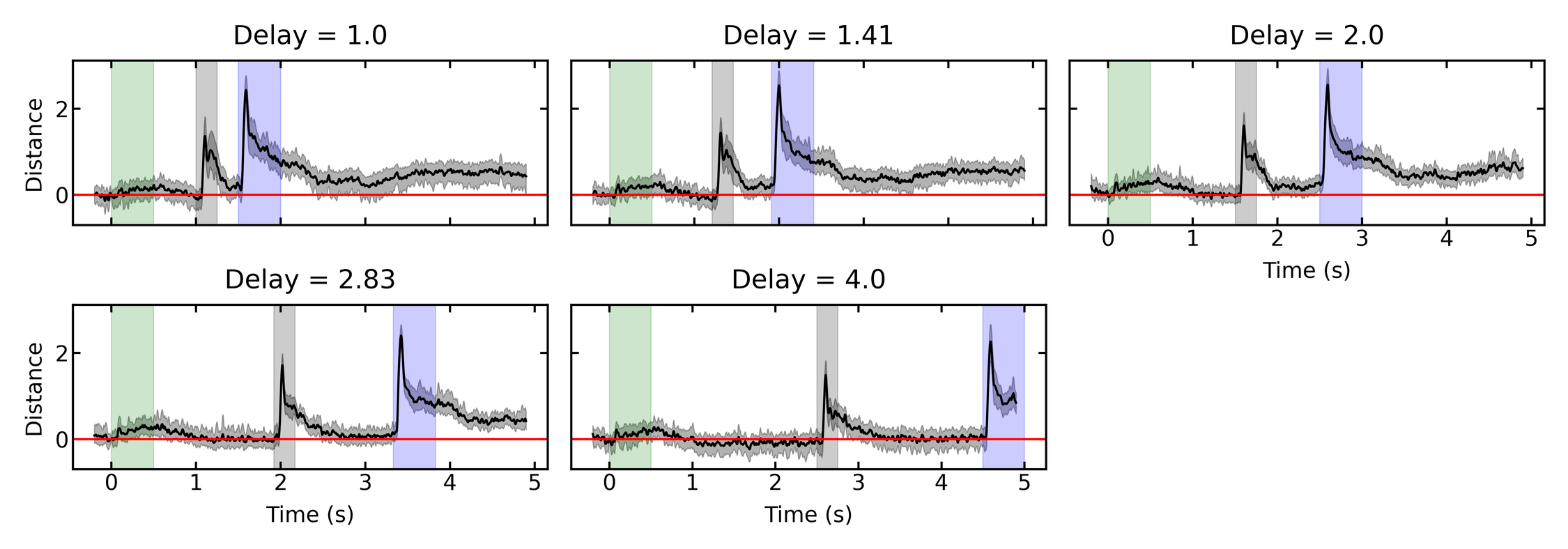

Supplement: S4 Fig — (TIF) [file pcbi.1010776.s004.tif]

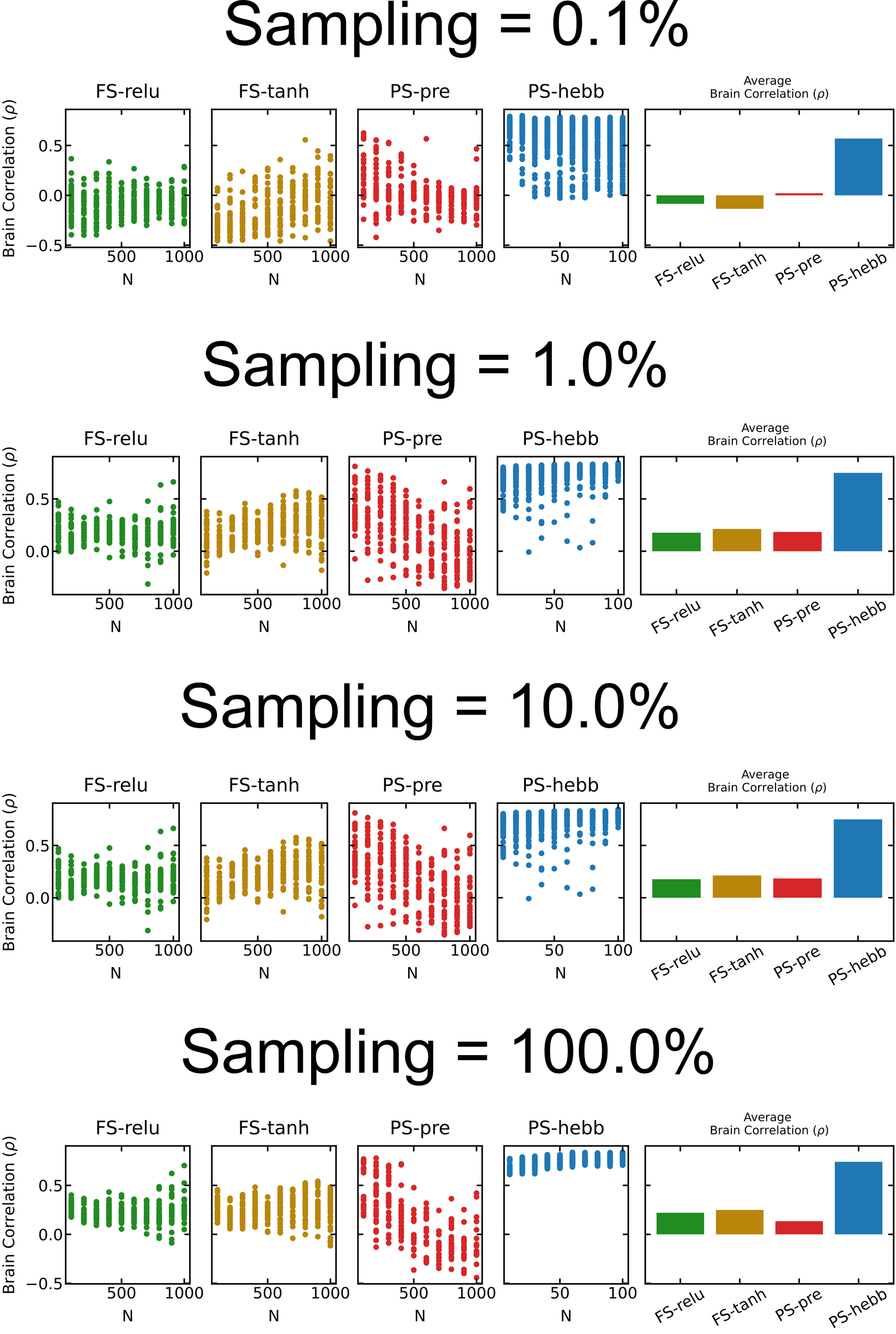

Supplement: S5 Fig — The qualitative result stays the same (PS-hebb is more brain-like on average), but the particular correlation values change. (TIF) [file pcbi.1010776.s005.tif]

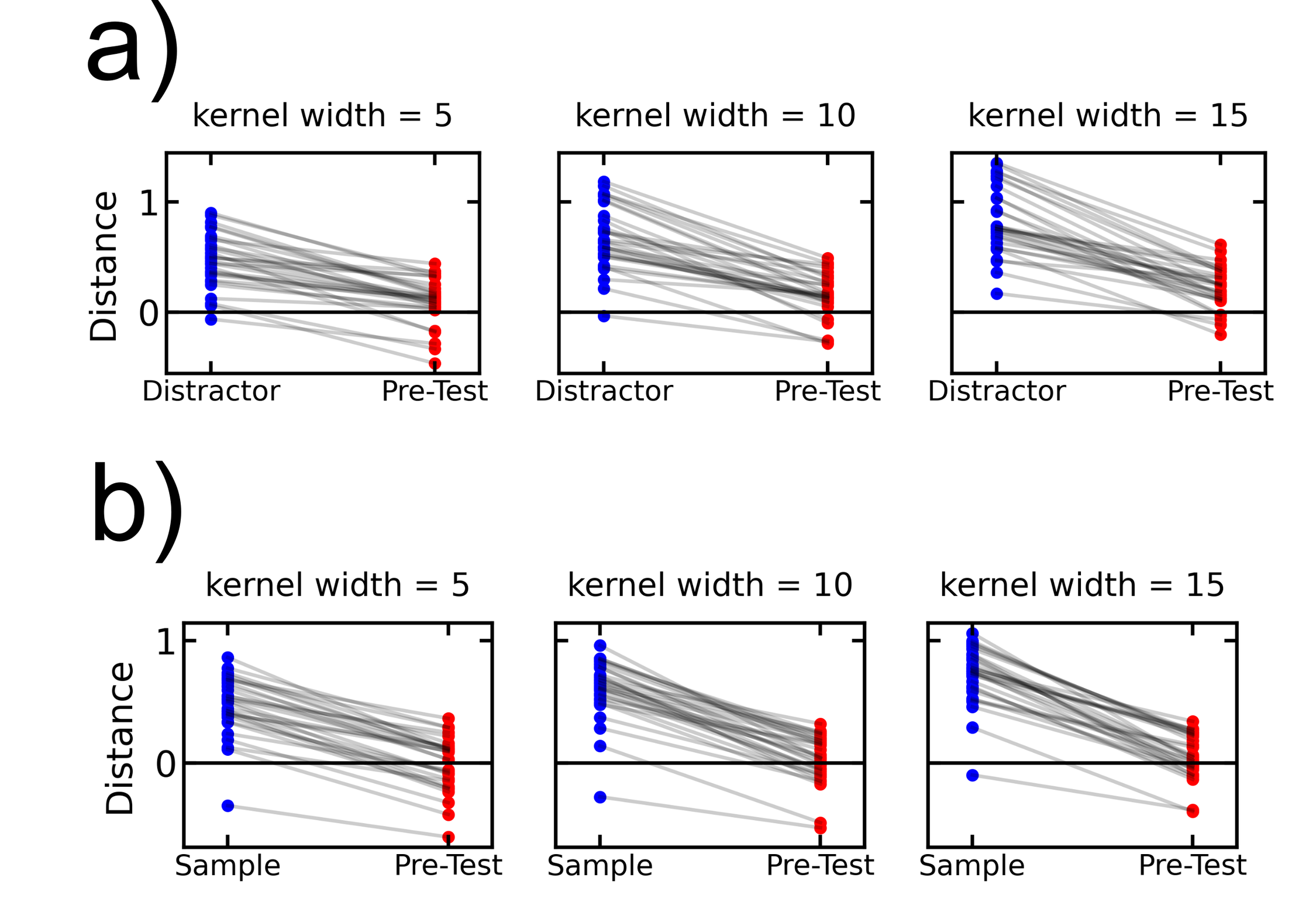

Supplement: S6 Fig — In all plots, each pair of dots correspond to averages during two time epochs for a particular session and delay time. In both cases the Pre-Test epoch used was 100ms preceding test time. All comparisons were statistically significant (p < 1e-3, Wilcoxon signed-rank test). a) Average distance between neural trajectories for different sample IDs for different smoothing parameters. Comparison is between Sample epoch (blue) and Pre-Test epoch (red). b) Average distance between neural trajectories for distracted and non-distracted trials for different smoothing parameters. Comparison is between Distractor epoch (blue) and Pre-Test epoch (red). (TIF) [file pcbi.1010776.s006.tif]

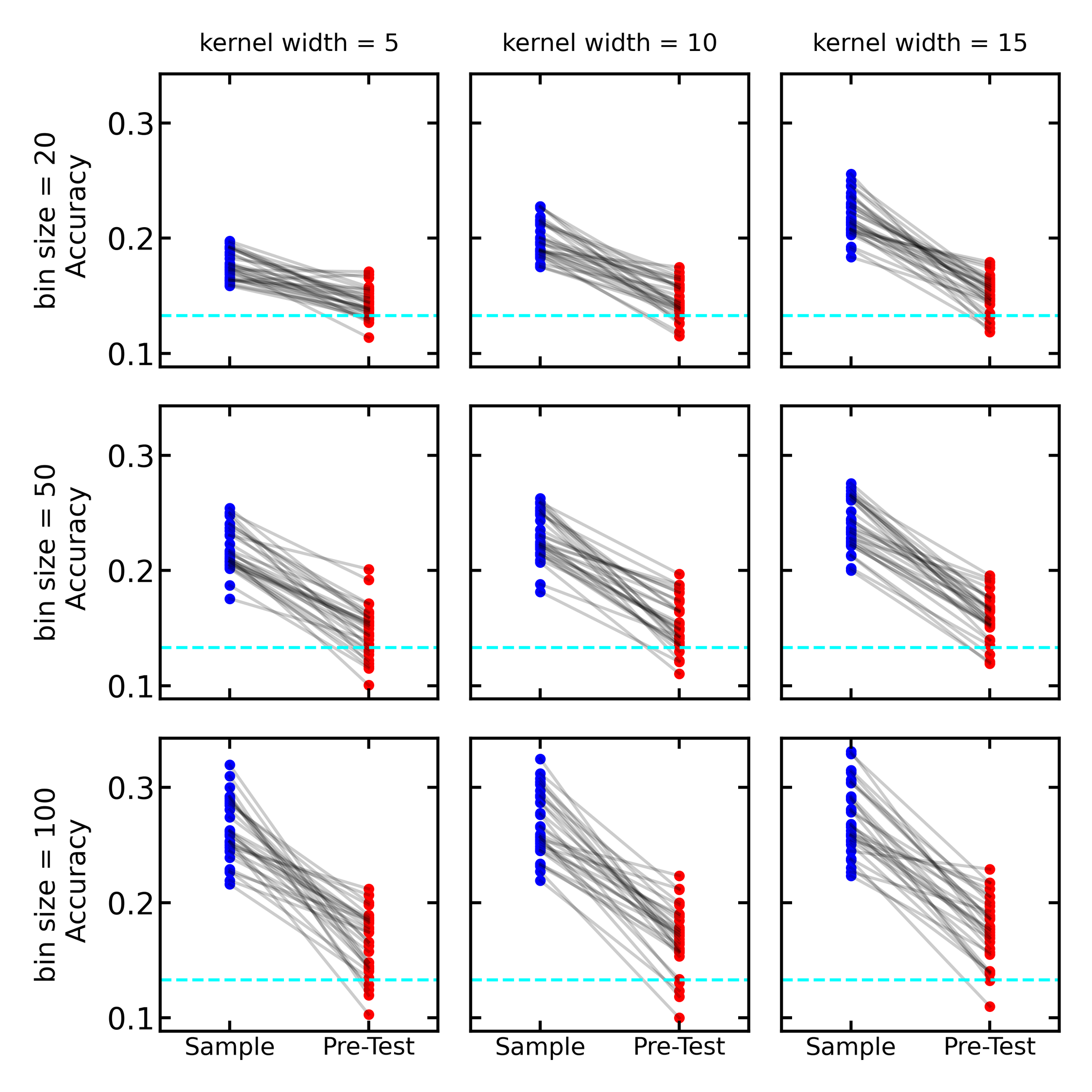

Supplement: S7 Fig — Comparison is between Sample epoch (blue) and Pre-Test epoch (red). In all plots, each pair of dots correspond to averages during two time epochs for a particular session and delay time. The Pre-Test epoch used was 100ms preceding test time. All comparisons were statistically significant (p < 1e-3, Wilcoxon signed-rank test). (TIF) [file pcbi.1010776.s007.tif]

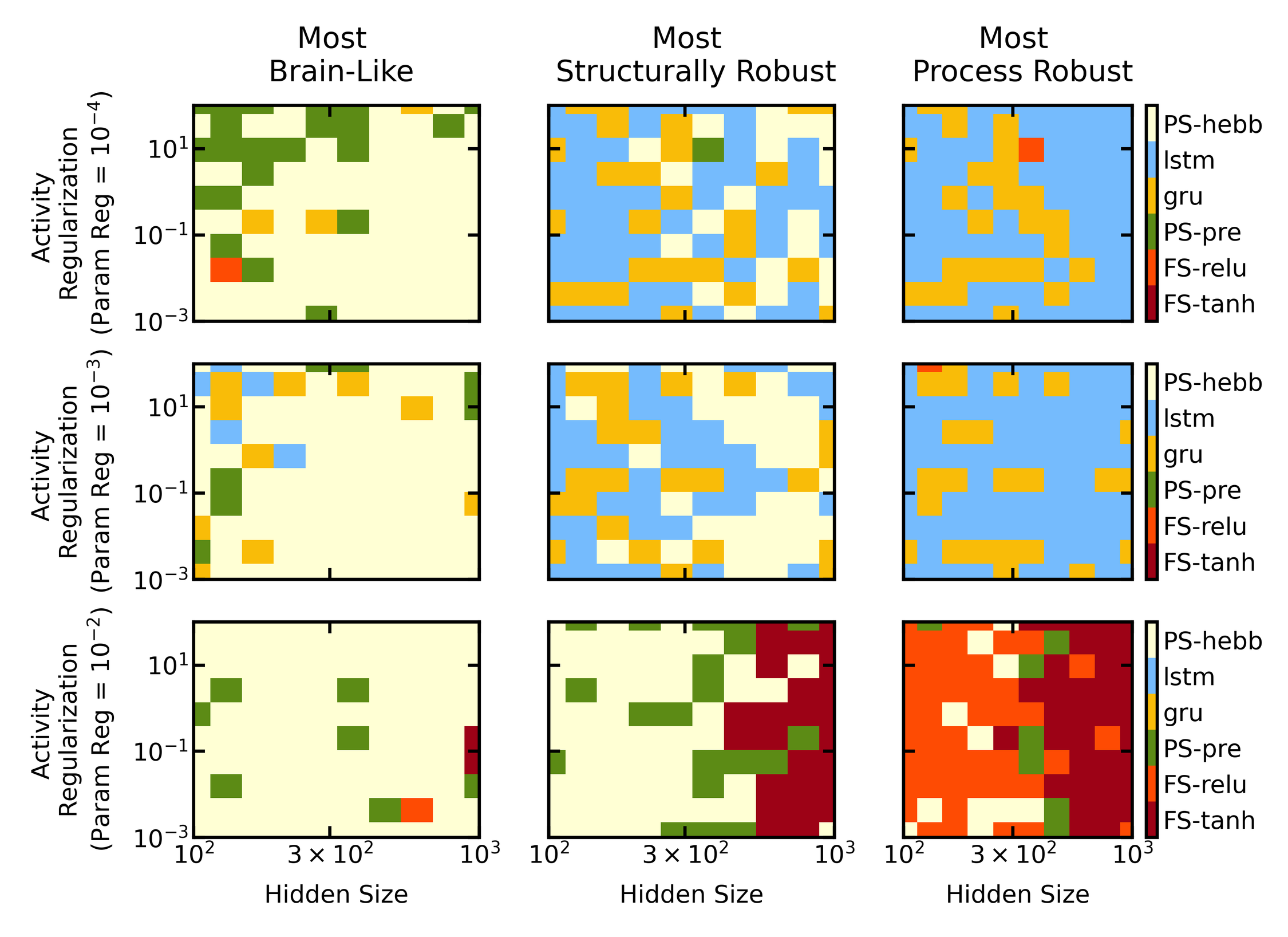

Supplement: S8 Fig — (TIF) [file pcbi.1010776.s008.tif]
